# Mapping of microRNAs related to cervical cancer in Latin American human genomic variants

**DOI:** 10.12688/f1000research.10138.2

**Published:** 2018-12-05

**Authors:** Milena Guerrero Flórez, Olivia Alexandra Guerrero Gómez, Jaqueline Mena Huertas, María Clara Yépez Chamorro

**Affiliations:** 1 Department of Biology, University of Nariño, Pasto, Nariño, Colombia; 2 Department of Biology, Center for Health Studies at the University of Nariño (CESUN), University of Nariño, Pasto, Nariño, Colombia

**Keywords:** cervical cancer, HPV, HPV integration sites, microRNAs, miRNAs, secondary structure, human genome variants, bioinformatics tools

## Abstract

**Background**: MicroRNAs are related to human cancers, including cervical cancer (CC) caused by HPV. In 2018, approximately 56.075 cases and 28.252 deaths from this cancer were registered in Latin America and the Caribbean according to GLOBOCAN reports. The main molecular mechanism of HPV in CC is related to integration of viral DNA into the hosts’ genome. However, the different variants in the human genome can result in different integration mechanisms, specifically involving microRNAs (miRNAs).

**Methods**: The miRNAs associated with CC were obtained from literature, the miRNA sequences and four human genome variants (HGV) from Latin American populations were obtained from miRBase and 1000 Genomes Browser, respectively. HPV integration sites near cell cycle regulatory genes were identified. miRNAs were mapped on HGV. miRSNPs were identified in the miRNA sequences located at HPV integration sites on the Latin American HGV.

**Results**: Two hundred seventy-two miRNAs associated with CC were identified in 139 reports from different geographic locations. By mapping with Blast-Like Alignment Tool (BLAT), 2028 binding sites were identified from these miRNAs on the human genome (version GRCh38/hg38); 42 miRNAs were located on unique integration sites; and miR-5095, miR-548c-5p and miR-548d-5p were involved with multiple genes related to the cell cycle. Thirty-seven miRNAs were mapped on the Latin American HGV (PUR, MXL, CLM and PEL), but only miR-11-3p, miR-31-3p, miR-107, miR-133a-3p, miR-133a-5p, miR-133b, miR-215-5p, miR-491-3p, miR-548d-5p and miR-944 were conserved.

**Conclusions**: Ten miRNAs were conserved in the four HGV. In the remaining 27 miRNAs, substitutions, deletions or insertions were observed. These variation patterns can imply differentiated mechanisms towards each genomic variant in human populations because of specific genomic patterns and geographic features. These findings may help in determining susceptibility for CC development. Further identification of cellular genes and signalling pathways involved in CC progression could lead new therapeutic strategies based on miRNAs.

## Introduction

Cervical cancer (CC) is the second most common malignancy in women worldwide. According to GLOBOCAN reports, approximately 569.847 women are diagnosed with CC and 311.365 die from it each year ^1^. Infection by human papillomavirus (HPV) has been recognized as the major risk factor in this pathology ^2, 3^, but the virus presence is not the main cause for the development of this cancer ^4, 5^. Viral DNA integration into the host cell genome is considered a conducive factor for cervical intraepithelial neoplasia (CIN) to develop into CC ^5– 7^.

Numerous microRNAs (miRNAs) have been identified in proximity to HPV integration sites ^8, 9^. miRNAs are a class of small (18 to 26 nucleotides length), noncoding, evolutionarily conserved RNAs that are processed from longer transcripts known as pre-miRNAs (60 to 100 nucleotides in length) ^10^. They are located on regions known as fragile sites and distributed in intergenic, intronic and exonic segments of the human genome involved in cancer ^11, 12^. Functionally, miRNAs has been recognized to participate in multiple cellular processes, including development, morphogenesis and carcinogenesis due to they regulate post-transcriptional expression levels of up to 60% of total protein-encoding genes by binding their seed sequences (2–8 nucleotides length). The 5'-UTR end of the miRNA seed sequence is complementary to the 3'-UTR end of the target mRNAs ^13^. This recognition event according to its length can affect the expression of important regulatory genes. Deregulation of genes such as tumour suppressor genes and oncogenes can lead to cancer development, including CC ^14– 16^.

Human genome variants generate different patterns of miRNA deregulation ^17^, which can contribute to cancer development susceptibility, treatment efficacy and patient prognosis ^18– 20^. 99% of the human genome is genetically identical, and the remaining 1% is responsible for all human diversity. miRNAs represent a major part of this genetic variation ^21^. miRSNPs (single nucleotide polymorphisms in miRNAs) are human polymorphisms at or near predicted miRNA target sites ^22^. The occurrence of miRSNPs can influence miRNA functionality on all levels, including transcription, maturation, and mRNA target binding.

Knowledge on miRNAs related to CC development in human genome variants from Latin American populations is scarce. Thus, in this study, we mapped miRNAs associated with CC in human genome variants obtained from Colombia, Mexico, Peru and Puerto Rico. Complete genomes were included in this study. Additionally, the relationships between HPV integration sites, genes close to these sites, mapping profiles and mutation patterns for each of the miRNAs were estimated for each of the genome sequences. The objective of this research was to analyse how genetic variation of CC-associated miRNAs identified in previously reported HPV integration sites affects cell cycle regulatory genes in human genomic variants from Latin America.

## Methods

### miRNA sequences associated with cervical cancer

Two hundred and seventy-two miRNAs associated with CC were selected as described in the systematic review published by Guerrero & Guerrero ^23^. With the information contained in miRBase ^24– 26^, miRNAMap ^27^ and miRNAstart, features such as length, chromosomal and genomic location of pre-miRNAs and mature miRNAs were analysed. The mature miRNA reference sequences were obtained in FASTA format from the miRBase database ( Dataset 1 ^28^).

### Latin American human genomic variants

Four human genome sequences were obtained from randomly selected female participants in the 1000 Genomes Project from Latin American populations ^22, 29^. Their codes were CLM (from Medellin in Colombia), MXL (from Los Angeles and of Mexican ancestry in the USA), PEL (from Lima in Peru) and PUR (from Puerto Rico). The control sequence was a variant that is phylogenetically distant to Latin American variants and identified with the code BEB (from Bangladesh and of Bengali ancestry). Access codes were obtained from the 1000 Genomes Project resources ^21, 30^. This information is summarized in Table 1.

**Table 1.** Accession numbers of the four Latin American human genome variants obtained from the NCBI 1000 genomes project.

TYPE SEQUENCE	NAME SEQUENCE	DATABASE	ACCESSION NUMBER
Genomic sequence	CLM	NCBI 1000 Genomes Project	HG01432
	MXL		NA19749
	PEL		HG01566
	PUR		HG00554
	BEB (Control)		HG03589

### Selection, identification and analysis of HPV integration sites near cell cycle regulatory genes

Viral insertion sites and nearby genes on the human genome were identified with the UCSC Genome Bioinformatics search engine ^31, 32^. To select HPV integration sites, a literature search was conducted in three databases ( PubMed, Science Direct and Springer link) using the terms: "HPV Integration sites AND Cervical Cancer". Positions of viral insertion sites and cellular genes close to these sites in the human genome were identified using the search engine tools available at UCSC Genome Browser on Human Dec. 2013 (GRCh38/hg38) Assembly: (a) search bar; (b) zoom in; (c) zoom out; (d) Mapping and Sequencing, chromosome band ( *full*); and (e) Genes and Gene Predictions, GENCODE v24 ( *full*) and NCBI RefSeq ( *full*) ^31^. To establish possible functional relationships with the development of CC, it was done by genes functional annotation described by UniProt ^33, 34^.

### Mapping miRNAs and chromosomal locations on the human genome

According to Xia *et al.* ^35^, the mature miRNA sequences are located in regions with pre-miRNA secondary structure complementarity (3' and 5'). In total, 445 miRNA sequences were analysed. The Blast-Like Alignment Tool (BLAT) available on the UCSC Genome Bioinformatics website was used for mapping the miRNAs associated with the full human genome with the following default parameters: (a) genome, human; (b) assembly, Dec. 2013 ( GRCh38/hg38); (c) query type, DNA; (d) sort output, query; and (e) score and output, hyperlinks. A matrix of chromosomal location data was built with Microsoft Excel 2013 (‘Matrix of data’ in Dataset 2 ^36^). From this matrix, the miRNAs over HPV integration sites were manually identified.

### Identification of miRNAs in Latin American human genomic variants

**Figure 1.**
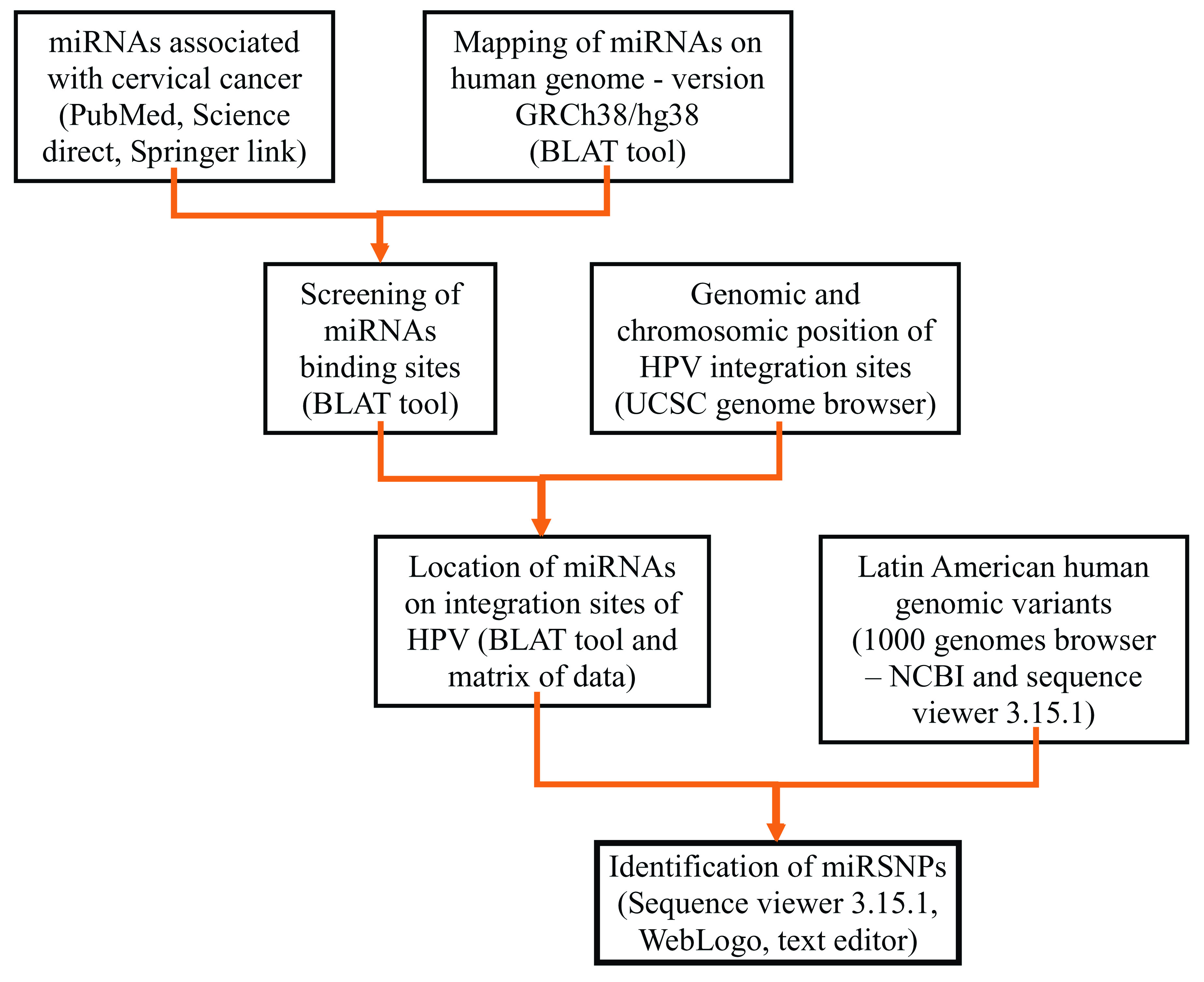
Bioinformatic workflow for mapping of miRNAs related to CC on Latin American human genomic variants.

To identify miRNA mutations in the four Latin American human genome variants, the available tools, including ideogram view, subjects and exon navigator, in the NCBI 1000 Genomes Browser (Phase 3, version 3.7) were used. The code for each female genetic variant selection (Colombia, Mexico, Peru, Puerto Rico and Bangladesh) was inserted and the sequence of each miRNA identified in viral integration sites was introduced and the mapped nucleotide positions were selected. Using WebLogo 3 ^37^, logos were created to view the nucleotide differences. The bioinformatics workflow is summarized in Figure 1.

Dataset 1. The mature miRNA reference sequences were obtained in FASTA format from the miRBase database http://dx.doi.org/10.5256/f1000research.10138.d164732
Copyright: © 2018 Guerrero Flórez M et al. 2018 Data associated with the article are available under the terms of the Creative Commons Zero "No rights reserved" data waiver (CC0 1.0 Public domain dedication).

Dataset 2. Matrix of data containing all the necessary components for the validation of data on CC-associated miRNAs in HPV integration sites in Latin American human genomic variants https://doi.org/10.5256/f1000research.10138.d217286
Copyright: © 2018 Guerrero Flórez M et al. 2018 Data associated with the article are available under the terms of the Creative Commons Zero "No rights reserved" data waiver (CC0 1.0 Public domain dedication).

## Results

### HPV integration sites and chromosomal distribution

**Figure 2.**
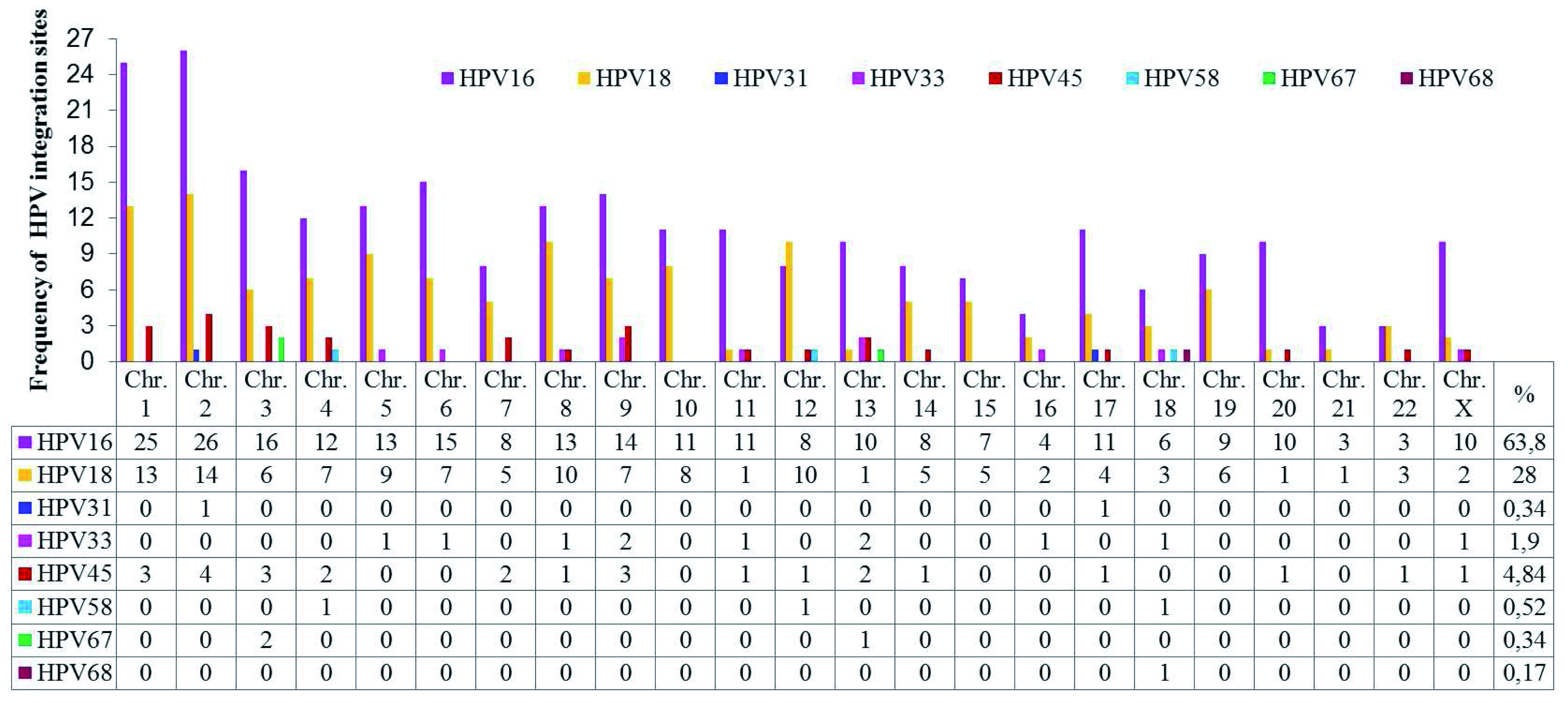
Chromosomal distribution of integration sites of HPV types (HPV 16, 18, 31, 33, 45, 58, 67 and 68) most frequently reported in the literature.

A total of 44 publications were identified between 1987 and 2015 related to HPV integration sites in the human genome. The most frequent types of HPV associated with CC were HPV-16 and HPV-18. Details of these articles are outlined in Supplementary File 1. Five hundred and sixty-eight integration sites for 8 types of HPV associated with different histological cervical conditions were identified, of which 63.84% were HPV-16 ( Figure 2 and ‘HPV integration sites’ in Dataset 2 ^36^).

HPV-16 and HPV-18 have integration sites on all human chromosomes. HPV-16 has more integration sites on chromosomes 2, 1, 3, 6, 9, 5, 8 and 4, while HPV-18 has more on chromosomes 2, 1, 8, 12, 5, 10, 4, 6 and 9. Some less frequently oncogenic HPV types have integration sites on specific chromosomes, such as HPV-45 on 2, 1, 3, 9, 4, 7 and 13; HPV-33 on 9, 13, 5, 6, 8, 11, 16, 18 and X; HPV-58 on 4, 12 and 18; HPV-31 on 2 and 17; HPV-67 on 4 and 13; and HPV-68 on chromosome 18. Chromosomes 1 and 2 displayed a higher number of viral insertion sites (41 and 45, respectively), while chromosomes 13 and 18 displayed insertion sites for 5 different HPV genotypes. The chromosomal loci with the highest numbers of HPV integration sites are presented in Table 2.

**Table 2.** Chromosomal loci with the highest numbers of HPV integration sites ^1^.

CHROMOSOMAL LOCUS	HPV INTEGRATION SITES	HPV TYPES
8q24.21	23	16,18,45
3q28 y 13q22.1	9	16,18,45
4q13.3	7	16,45
2q34	6	16,18
2q22.3 y 20p12.1	5	16,18
13q21 y 17q12	5	16

^1^Chromosomal bands that have more than 5 HPV integration sites.

### Analysis of HPV integration sites near cell cycle regulatory genes

**Figure 3.**
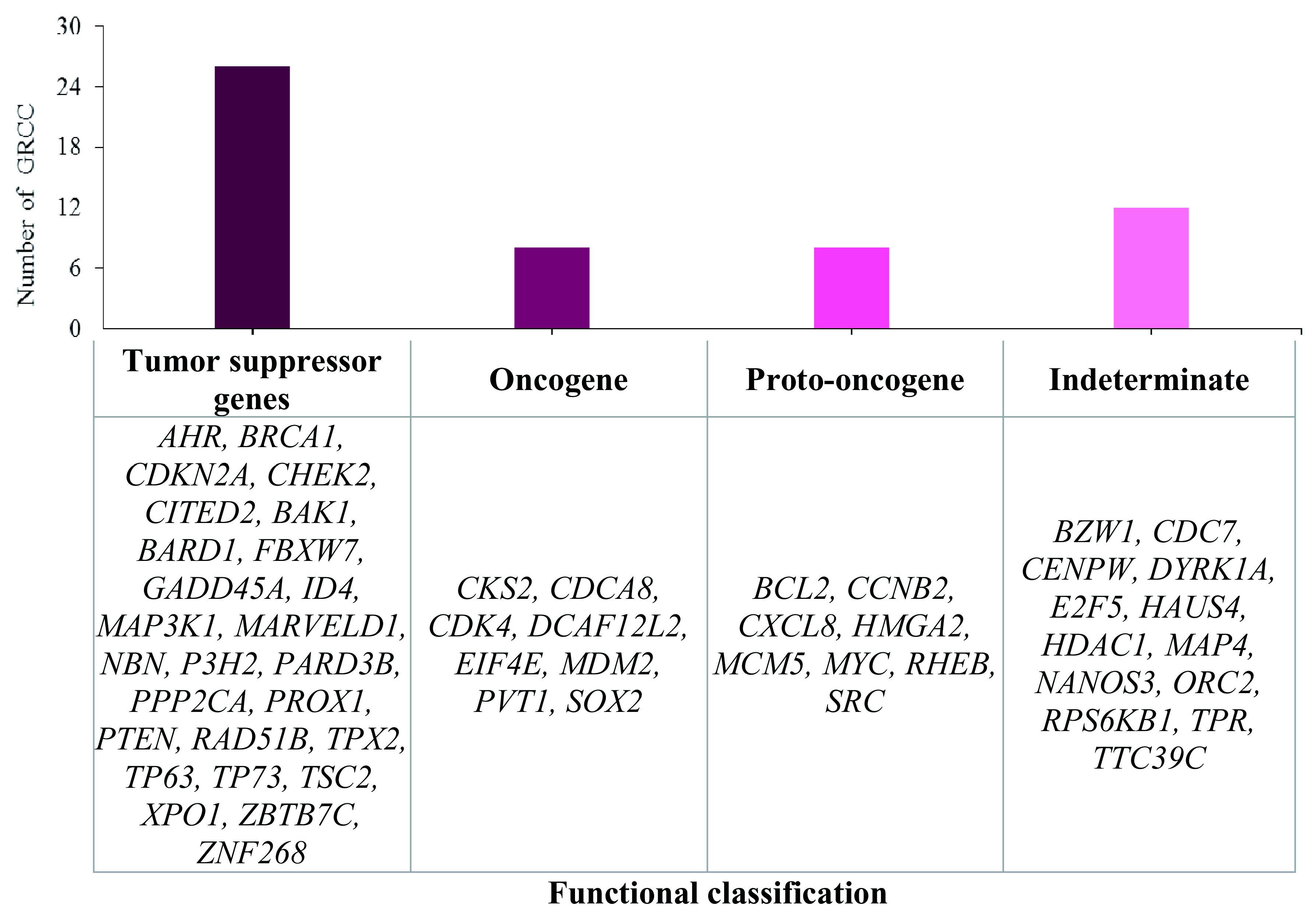
Functional classification of cellular genes in HPV integration sites (GRCC: cell cycle regulatory genes).

Information on the associated functions of genes located near HPV integration sites obtained from UniProt showed that 86.1% of the genes located in close proximity were involved in apoptosis, cell adhesion, cell differentiation, ion transport and metabolic processes. Fifty-four genes were involved in direct regulation of the cell cycle. Twenty-six of these were tumour suppressor genes, 8 were oncogenes, 8 were proto-oncogenes and 13 did not have a determined functionality in the development of this neoplasia ( Figure 3).

### Mapping miRNAs associated with cervical cancer

The 2028 miRNA binding sites associated with CC in the human genome were identified from BLAT mapping using previously identified miRNAs ^23^, including 432 sites previously reported in miRBase (‘Results of mapping with BLAT’ in Dataset 2 ^36^). These sites were located on both DNA strands (52.97% on the positive strand and 47.03% on the negative strand). 1881 binding sites were fully complementary (100% sequence identity) to miRNA sequences, while 1, 24, and 122 binding sites had 96.2%, 95.7% and 95.5% sequence identity, respectively.

miR-5095 was mapped onto 853 binding sites on 23 chromosomes. Four hundred and twenty-four mature miRNAs sequences (98.15%) mapped to one, two, three and even ten different binding sites. miR-522-5p and miR-523-5p binding sites mapped only a single chromosome (Chr. 19). Table 3 shows the chromosomal location and number of binding sites for each specific miRNA associated with CC.

**Table 3.** Chromosomal location and frequency of miRNA binding sites associated with CC ^1^.

miRNA ASSOCIATED WITH CC	miRNAs BINDING SITES	CHROMOSOMAL LOCATION
hsa-miR-5095	853	All chromosome
hsa-miR-548c-5p	194	All, except 9
hsa-miR-548d-5p	188	All, except X, Y
hsa-miR-548b-5p	87	All, except 3, 4, 5, 6, X, Y
hsa-miR-574-5p	62	All, except 16, 21, Y
hsa-miR-576-3p	15	4, 5, 8, 9, 12, 13, 15, 18, 22, X
hsa-miR-548c-3p	13	2, 4, 5, 7, 8, 13, 14, X, Y
hsa-miR-1273g-5p	11	1, 3, 7, 9, 10, 11, 13, 14, 15
hsa-miR-95-5p	10	1, 2, 4, 6, 7, 13, X
hsa-miR-1244	9	2, 3, 5, 7, 12, 13, 14, 20
hsa-miR-545-3p	8	3, 5, 7, 10, 12, X
hsa-miR-378a-3p	7	3, 5, 10, 11, 14, 17, 18
hsa-miR-522-5p, -523-5p	7	19
hsa-miR-518f-5p	6	5, 19
hsa-miR-545-5p	6	2, 3, 5, 14, 17, X
hsa-miR-151a-5p	5	1, 4, 8, 19, X
hsa-miR-339-5p	5	5, 7, 20, 22
hsa-miR-603	4	10, 13, 14, 16
hsa-miR-7-5p	4	9, 10, 15, 19
hsa-miR-584-5p	4	4, 5, 9, 19

^1^miRNAs associated with CC mapped more than 4 positions.

The distribution of the 2028 binding sites was not homogeneous along the human genome. 41% of the total binding sites were identified on chromosomes 1, 19, 5, 2, 3, 14, 7 and X. Although the number of miRNA binding sites correlated with the size of each chromosome, some short chromosomes, such as 19 and X, had more miRNA binding sites when compared to other larger chromosomes ( Table 4).

**Table 4.** Chromosomal distribution of binding sites identified in miRNAs associated with CC.

CHR. ^1^	NUMBER OF miRNAs BINDING SITES	(%)
**1**	175	8,63
**2**	108	5,33
**3**	106	5,23
**4**	89	4,39
**5**	111	5,47
**6**	87	4,29
**7**	103	5,08
**8**	81	3,99
**9**	79	3,90
**10**	92	4,54
**11**	93	4,59
**12**	93	4,59
13	71	3,50
14	106	5,23
15	66	3,25
16	81	3,99
17	94	4,64
18	57	2,81
19	131	6,46
20	42	2,07
21	27	1,33
22	29	1,43
X	100	4,93

^1^ **CHR**= Chromosome.

14.89% (302) of binding sites grouped into the following 19 specific chromosomal locations: (1) 19q13.42 (51 sites/14 miRNAs), (2) 14q32.31 (34 sites/16 miRNAs), (3) 13q31.3 (16 sites/11 miRNAs), (4) 14q32.2 (16 sites/9 miRNAs), (5) 4q25 (16 sites/7 miRNAs), (6) 20q13.33 (15 sites/7 miRNAs), (7) 16p13.3 (15 sites/4 miRNAs), (8) Xq26.2 (14 sites/8 miRNAs), (9) 7q22.1 (14 sites/6 miRNAs) and (10) 1p31.3 (14 sites/6 miRNAs). The remaining 9 chromosomal locations contained between 10 and 13 binding sites ( Supplementary File 2). 92% (1865/2028) of the binding sites were distributed into 250 groups along the human genome; the remaining 8% (163/2028) of binding sites for various miRNAs including miR-5095 were distributed along the human genome without being distributed into any groups.

**Figure 4.**
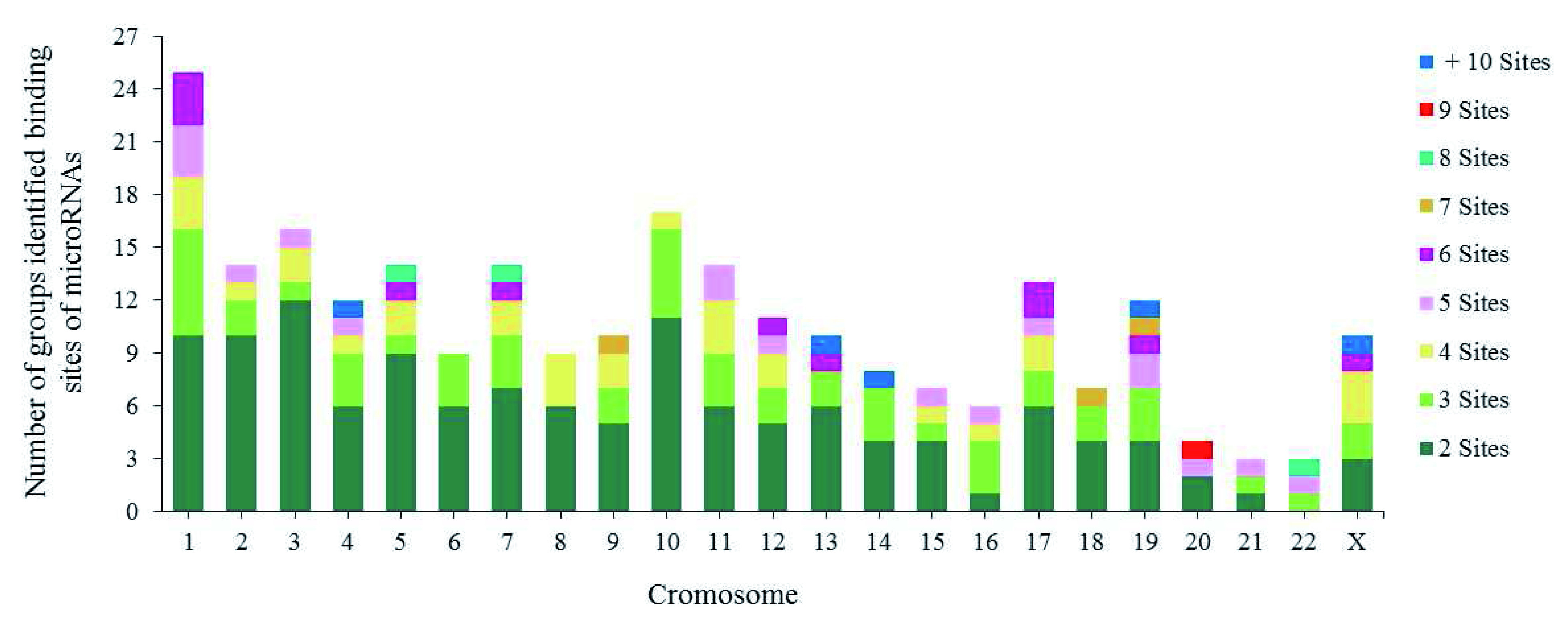
Chromosomic distribution of groups identified binding sites of miRNAs.

Each group contains between 2 and 7 miRNA binding sites, although some groups contain between 8 and 16 ( Figure 4). The majority of the groups are located on chromosomes 1, 2, 3, 5, 10 and 11. The biggest groups are located on chromosome 19, with 51 binding sites for 25 miRNAs involved in CC development.

**Figure 5.**
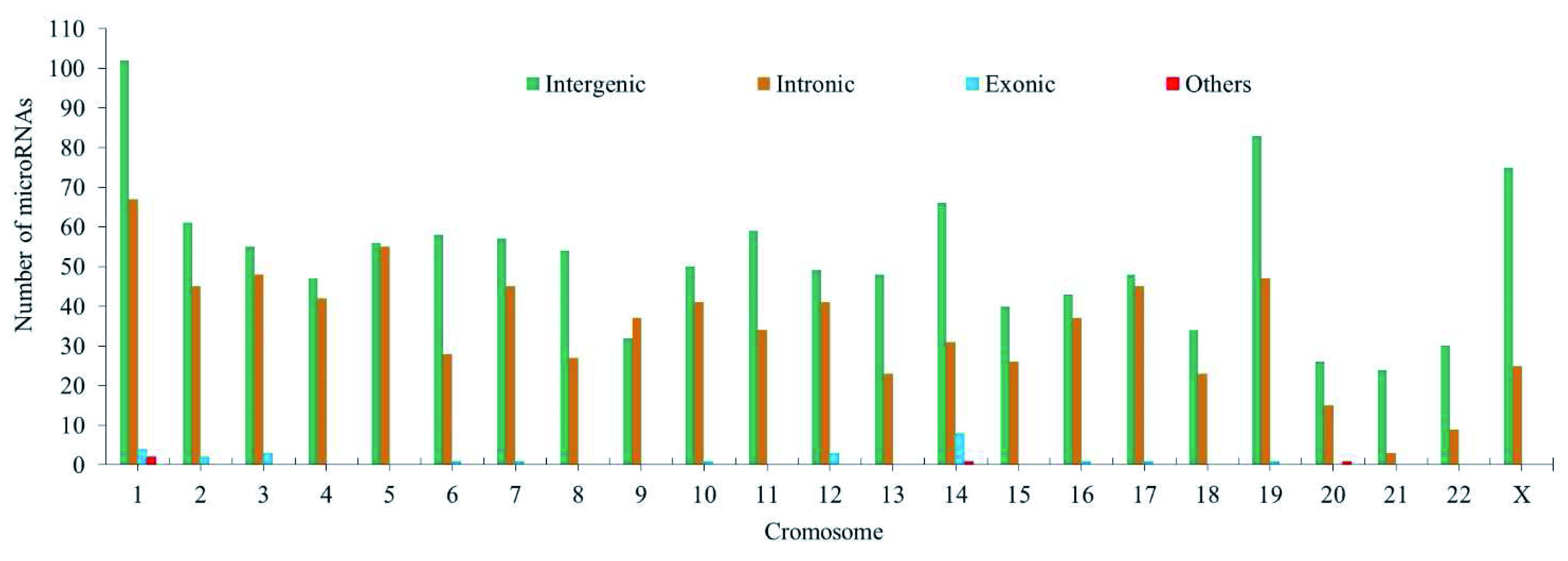
Numeric variation of miRNAs associated with the development of CC in different genomic locations (intergenic, intronic and exonic) per chromosome.

58.8% of miRNA binding sites associated with CC (1194 binding sites) are located in intergenic regions, 39.65% (804 binding sites) in intronic regions, 1.28% (26 binding sites) in exonic regions and 0.19% (4 binding sites) between intronic and exonic regions (mixed miRNAs). Figure 5 shows the variation in the number of intergenic, exonic and intronic miRNAs associated with CC.

### miRNA identification in selected HPV integration sites

Thirty-eight integration sites were found for six types of oncogenic HPV (HPV-16, -18, -33, -45, -58 and -68) in miRNA binding sites and cell cycle regulatory genes associated with CC ( Table 5). The largest number of HPV integration sites was found for miR-5095 (33 sites), followed by miR-548c-5p (11 sites) and miR-548d-5p (11 sites) ( Table 5). In 14 integration sites, no miRNA binding sites were detected. The highest number of miRNA binding sites was found in chromosome regions 18q11.2 and 19p13.12 ( Supplementary File 2).

**Table 5.** miRNAs in HPV integration sites and their correlation with cell cycle regulatory genes.

HPV TYPES	HPV INTEGRATION SITES	miRNAs PRESENT AT HPV INTEGRATION SITES ^1^	CELLULAR GENES ^2^	CL. ^3^
18	1p22.2	miR-548c-5p (-)	*CDC7* (+)	--
18	1p31.2	-	*GADD45A* (+)	ST
16	1p34.1	-	*PLK3* (+)	--
16	1p34.3	miR-5095 (3; -,-,+), -548b-5p (-), -548c-5p (2, -,-), -548d-5p (-)	*CDCA8* (+)	OG
16	1q25	-	*TPR* (-)	--
16	1q36.32	-	*TP73* (+)	ST
16,18	1q41	miR-5095 (2,+,+), -194-5p (-), -215-3p (-), -215-5p (-), -548b-5p (-)	*PROX1* (+)	ST
18	2p15	miR-5095 (-)	*XPO1* (-)	ST
16	2q33.1	miR-152-5p(-), -548d-5p(-)	*ORC2* (-)	--
			*BZW1* (+)	--
16	2q33.3	miR-5095 (+)	*PARD3B* (+)	ST
16	2q34	miR-5095 (-)	*BARD1* (-)	ST
16	3p21.31	miR-5095 (3;-,+,+), -191-3p (-), -191-5p (-), -425-3p (-), -425-5p (-)	*MAP4* (-)	--
16	3q26.33	miR-5095 (2; -,+)	*SOX2* (+)	OG
16	3q28	miR-5095 (-), -944 (+), -28-3p (+), -28-5p (+)	*P3H2* (-)	ST
			*TP63* (+)	ST
16, 45	4q13.3	-	*CXCL8* (+)	PO
16	4q23	-	*EIF4E* (-)	OG
16	4q31.21	miR-548c-5p (+)	*FBXW7* (-)	ST
16	5q11.2	miR-5095 (3; -,-,+), -449a (-), -449b-3p (-), -449b-5p (-), -548c-3p (+), -548d-5p (+), -581 (-)	*MAP3K1* (+)	ST
16	5q31.1	miR-5095 (-)	*PPP2CA* (-)	ST
16	6p21.31	miR-5095 (+)	*BAK1* (-)	ST
16	6p22.3	miR-5095 (4; -,-,+,+), -548c-5p (+), -548d-5p (2; +,+)	*ID4* (+)	ST
16	6q22.32	-	*CENPW* (+)	--
16	6q23.3	miR-5095 (3; -,+,+)	*CITED2* (-)	ST
16	7p21.1	-	*AHR* (+)	ST
18	7q36.2	miR-5095 (-)	*RHEB* (-)	PO
18	8q21.2	-	*E2F5* (+)	--
16, 18	8q21.3	-	*NBN* (-)	ST
16, 18, 45	8q24.21	miR-5095 (-), -548d-5p (-)	*MYC* (+)	PO
16	8q24.21	miR-5095 (-), -548d-5p (-)	*PVT1* (+)	OG
18	9p21.3	miR-5095 (+), -31-3p (-), -31-5p (-), -491-3p (+), -491-5p (+)	*CDKN2A* (-)	ST
16	9q22.2	miR-5095 (+), -576-3p (2; +,+)	*CKS2* (+)	OG
16, 18	10q23.31	miR-5095 (-), -107 (-), -103a-3p (-), -548b-5p (2; -,-), -548d-5p (2; -,-)	*PTEN* (+)	ST
16	10q24.2	miR-5095 (-), -1287-5p (-)	*MARVELD1* (+)	ST
16	12q14.3	miR-574-5p (-)	*CDK4* (-)	OG
			*MDM2* (+)	OG
18	12q15	-	*HMGA2* (+)	PO
58	12q24.33	-	*ZNF268* (+)	ST
18	14q11.2	miR-5095 (+), -548c-3p (+), -574-5p (+)	*HAUS4* (-)	--
18, 45	14q24.1	miR-5095 (2, -,+), -548c-5p (+)	*RAD51B* (+)	ST
18	15q21.3	miR-5095 (2; -,+), -574-5p (-)	*CCNB2* (+)	PO
16	16p13.3	miR-5095 (12; (7 -, 5+,)), -548c-5p (+), -572 (-), -940 (+)	*TSC2* (+)	ST
16	17q21.31	miR-5095 (3; -,+,+)	*BRCA1* (-)	ST
33	18q11.2	miR-5095 (-), -1-3p (-), -133a-3p, -133a-5p (-), -133b, -378a-3p (+), -548b-5p (-), -548d-5p (-)	*TTC39C* (+)	--
68	18q21.1	miR-5095 (3; -,+,+), -548c-5p (+), -548d-5p (+), -574-5p(+)	*ZBTB7C* (-)	ST
18	18q21.33	miR-5095 (-), -548b-5p (+), -548c-5p (-), -548d-5p (+)	*BCL2* (-)	PO
16	19p13.12	miR-5095 (-), -23a-3p (-), -23a-5p (-), -27a-3p (-), -27a-5p (-), -181c-3p (+), -181c-5p (+), -584-5p (+)	*NANOS3* (+)	--
16	20q11.21	-	*TPX2* (+)	ST
16	20q13.2	miR-5095 (-)	*SRC* (+)	PO
16	21q22.13	miR-5095(+), -548d-5p (-)	*DYRK1A* (+)	--
16	22q12.1	miR-548c-5p (+)	*CHEK2* (-)	ST
16, 18, 45	22q13.1	miR-5095 (2, -,-)	*MCM5* (+)	PO
16	Xq25	miR-5095 (-), -574-5p (-)	*DCAF12L2* (-)	OG

^1^In parentheses, the number of binding sites of miRNAs and DNA chain where miRNAs are located. ^2^In parentheses DNA chain where the cell cycle regulatory genes are located. ^3^Cl: Classification of cellular genes; ST: tumor suppressors; OG: Oncogenes; PO: Proto-oncogenes.

**Figure 6.**
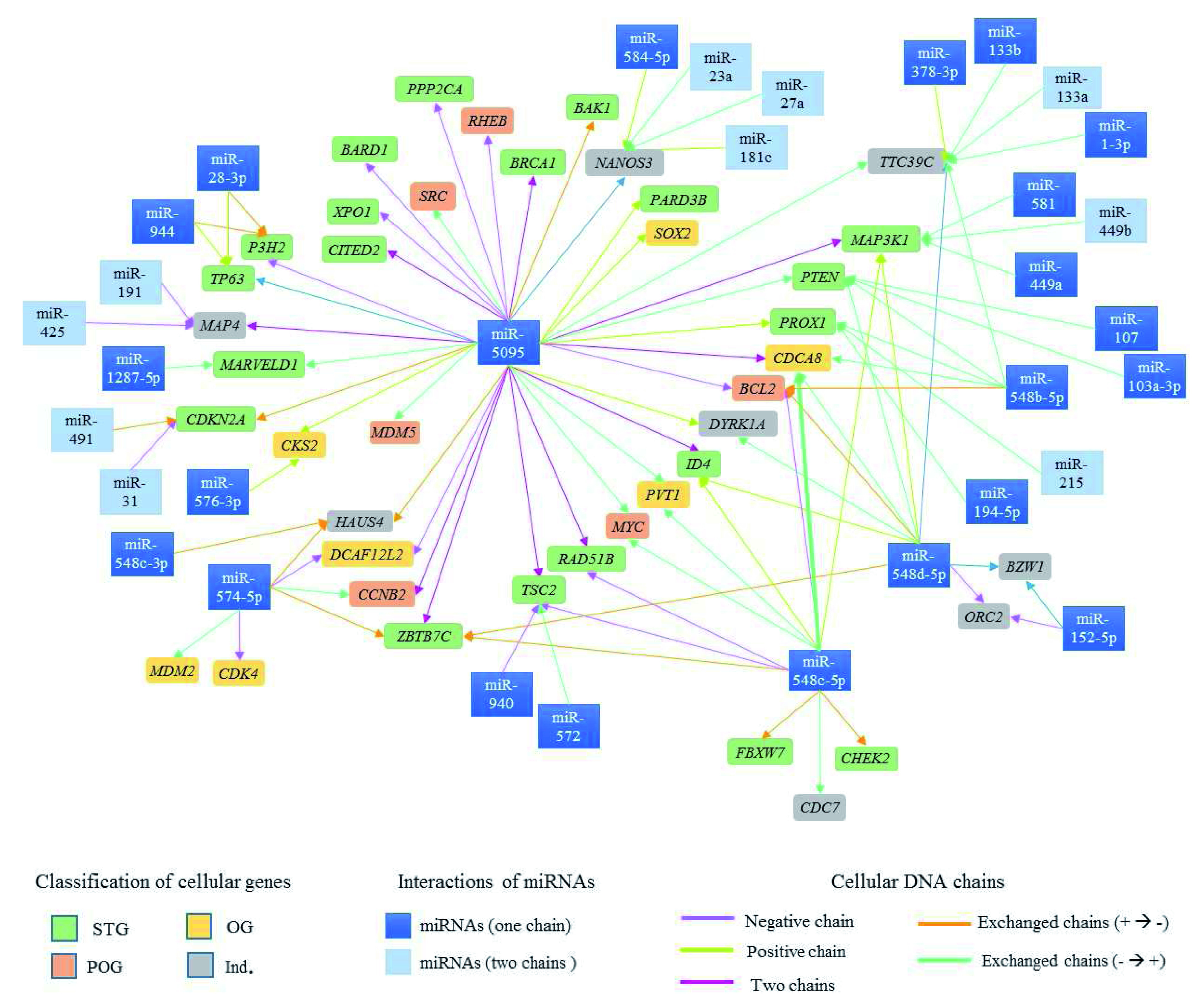
Possible network of interactions between miRNAs associated with development of CC and cell cycle regulatory genes present at HPV integration sites. The cell cycle regulatory genes in rectangles of various colors are presented, depends on their classification (ST - 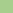, OG - 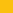, POG - 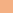 e IND - 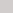). The arrows represent the interactions between miRNAs and genes involved in cell cycle regulation, dates color depends on the DNA chain where miRNAs and cell cycle regulatory genes are located.

Ninety-six possible interactions were identified between 37 mature miRNAs associated with CC and 42 cell cycle regulatory genes located in proximity to the viral insertion sites. The network of interactions is presented in Figure 6. 35.42% of the interactions involved miR-5095, 12.5% involved miR-548c-5p and 12.5% miR-548d-5p.

38.1% of genes identified in HPV integration sites have binding sites for a single miRNA, and 61.9% have binding sites for more than two miRNAs. Table 6 displays genes with more than five miRNA binding sites.

**Table 6.** Gene associated a more five binding sites of miRNAs.

NUMBER OF miRNA BINDING SITES	miRNAs	GENE
5 sites	miR-103a-3p, -107, -548b-5p, -548d-5p and -5095	*PTEN*
	miR-194-5p, -215-3p, -215-5p, -548b-5p and -5095	*PROX1*
7 sites	miR-449a, -449b-3p, -449b-5p, -548c-3p, -548d-5p, -581 and -5095	MAP3K1
8 sites	miR-1-3p, -133a-3p, -133a-5p, -133b, -378a-3p, -548b-5p, -548d-5p and -5095	*TTC39C*
	miR-23a-3p, -23a-5p, -27a-3p, -27a-5p, -181c-3p, -181c-5p, -584-5p and -5095	*NANOS3*

A gene may have binding sites for both regions of complementarity (3' and 5') of a miRNA ^38^. In this study, we found that the TTC39C gene has binding sites for miR-133a-3p and miR-133a-5p and MAP3K1 has binding sites for miR-449b-3p and miR-449b-5p, though some mature sequences from one miRNA also showed binding sites to different genes ( Figure 6). As an example, the miR-548c-3p mature chain has binding sites in the HAUS4 gene as well as in the MAP3K1, CDCA8, BCL2, ID4, cMYC, RAD51B, TSC2, ZBTB7C, FBXW7, CHEK2 and CDC7 genes ( Figure 6).

### Identification of miRNAs on Latin American human genomic variants

**Figure 7.**
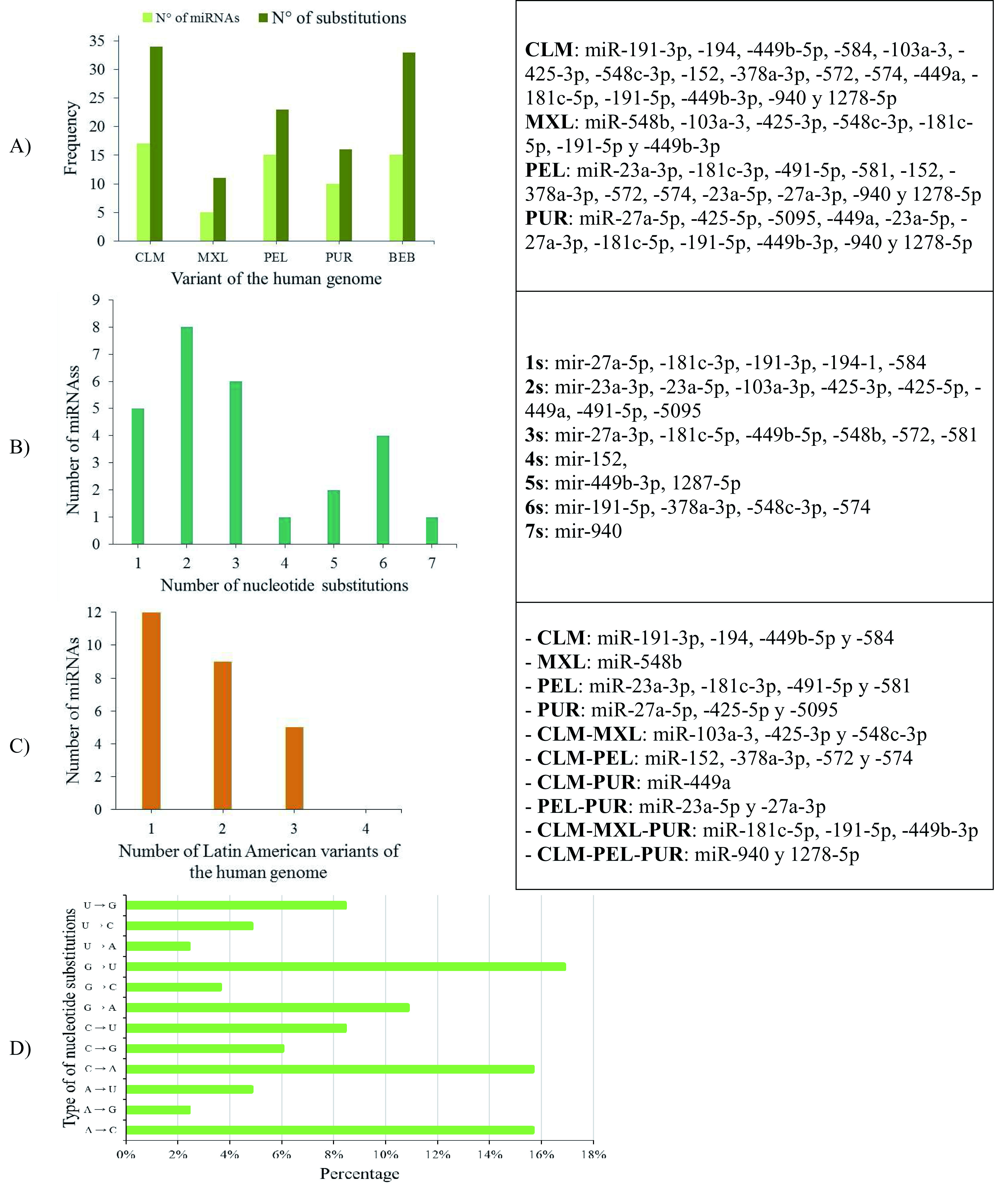
**A**) Number of miRNAs and nucleotide substitutions found in each human genomic variant; **B**) Number of miRNAs with between 1 and 7 nucleotide substitutions; **C**) Number of miRNAs with nucleotide substitutions in one, two or three genomic variants in the Latin American human genome, and **D**) Percentage of types of nucleotide substitutions in the miRNA sequences associated with CC in the selected human genome variants.

26.31% (10/42) of the miRNAs analysed (miR-11-3p, miR-31-3p, miR-107, miR-133a-3p, miR-133a-5p, miR-133b, miR-215-5p, miR-491-3p, miR-548d-5p and miR-944) were identical across the Latin American human genome variants, and 73.69% showed a genetic mutation (substitution or deletion of nucleotides) ( Figure 7, Panels A and B).

When mapping the sequences of these miRNAs to the selected Latin American human genome variants ( Supplementary File 3), 88 miRSNPs related to miRNAs or miRNA binding sites were identified on the Latin American variants compared with 33 on the reference variant. Twenty-one miRSNPs were located in the miRNA seed sequences of Latin American variants compared with 3 located in the reference variant. The most representative mapping results are shown in Table 6.

Types of nucleotide substitutions in the miRNA sequences associated with CC in the selected human genome variants showed that there were more frequent transversions than transitions and that the most frequent nucleotide substitutions were G→U (16.9%), followed by A→C (15.7%), C→A (15.7%) and G→A (10.8%) ( Figure 7).

Between one and 18 nucleotide deletions were detected in miR-27a-3p, miR-31-5p, miR-103a-3p, miR-191-3p, miR-215-3p and miR-574. The sequences of miR-28, miR-152, miR-548c-5p, miR-572 and miR-5095 only mapped to reference sequences (version GRCh38/hg38), but not to any of the Latin American human genomic variants. miR-152 did not map to the PUR variant ( Table 6).

Table 7 displays the nucleotide variations from human genome variants obtained from Colombia, Mexico, Peru and Puerto Rico and Bangladesh, which was the control variant.

**Table 7.** miRNAs identified in HPV integration sites, displaying the nucleotide variations in the selected Latin American human genome variants and the control variant. More data is available in Supplementary File 3.

HG ^1^	miRNAs IDENTIFIED IN HPV INTEGRATION SITES (Cromosomal location (Chain)) ^2^	
	hsa-mir-1-3p (18q11.2 (-))	hsa-mir-23a-3p (19p13.12 (-))
**CLM** **MXL** **PEL** **PUR** **BEB**	UGGAAUGUAAAGAAGUAUGUAU UGGAAUGUAAAGAAGUAUGUAU UGGAAUGUAAAGAAGUAUGUAU UGGAAUGUAAAGAAGUAUGUAU UGGAAUGUAAAGAAGUAUGUAU	AUC ACAUU GCCA GGGAUUUCC AUC ACAUU GCCA GGGAUUUCC AU A ACAUU GCA A GGGAUUUCC AUC ACAUU GCCA GGGAUUUCC AUC ACAU C GCCA GGGAUUUCC
	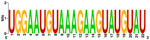	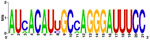
	**Conserved**	**Nucleotide substitution**
	hsa-mir-31-5p (9p21.3 (-))	hsa-mir-152 (17q21.32 (-))
**CLM** **MXL** **PEL** **PUR** **BEB**	AGGCAAGAUGCUGGCAU AGCU AGGCAAGAUGCUGGCAU AGCU AGGCAAGAUGCUGGCAU AGGCAAGAUGCUGGCAU AGCU AGGCAAGAUGCUGGCAU AGCU	C GG G UCUGUG C UA CACUCC GACU C GACU A GGU UCUGUGA UA CACU AC GACU A GGU UCUGU UG U G CACUC U GACU
	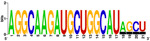	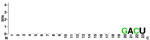
**|**	**Nucleotide deletion**	**Absence of the miRNA sequence**

^1^HG: Human genome; CLM: variant of Medellin, Colombia; MXL: Los Angeles with Mexican ancestry; PEL: Lima, Peru; PUR: of Puerto Rico; BEB: Bengali, Bangladesh. ^2^The size of each letter indicates the enrichment of each nucleotide in Latin American variants of the human genome, WebLogo displayed through the program..

## Discussion

### HPV integration sites

According to the literature, approximately 570 integration sites have been identified for eight oncogenic HPV types associated with CC ( Figure 2). HPV integration into cellular DNA and consequent deregulation of genes is considered a crucial step in cancer progression. Genotype HPV-16 is the most studied for its relationship with CC, as it is responsible for 70% of cases worldwide ^39^. This could be a consequence of the greater proportion of integration sites reported for this genotype. In contrast, low risk genotypes, such as HPV-45, -66 and -93 reported in Colombia, are frequent in CC ^40– 44^.

HPV integration into the host genome occurs in regions well-known as fragile sites, breakpoints or transcriptionally active regions ^45^. This integration induces functional alterations of cellular genes in close proximity ^12, 46– 48^. According to our results, the 8q24.21 chromosome region is the most affected by HPV integration. If we take into account that proto-oncogenes such as the MYC gene are located here ^49^(as displayed in Figure 3) and that MYC represents a family of genes overexpressed in several tumours including CC ^49– 51^, inhibition of MYC expression can induce cancer cell destruction ^50^. In this context, the MYC gene could be both a tumour biomarker and potential treatment target for several tumours ^51^ ( Table 2).

Chromosomes 1, 14, 19 and X contain significantly more mature miRNAs than others, and chromosome 18 contains fewer miRNAs. The 19q13.4 chromosome region contains the largest group of human miRNAs (known as the group of miRNAs on chromosome 19 "C19 MC"), with alterations in several that have been previously reported in cancer ^52^. Studies have reported associations between chromosome 1 and malignant transformation in cancers, including CC ^53^.

The 578 integration sites identified in eight HPV types associated with CC were located in cell cycle regulatory genes, including the tumour suppressor genes TP73, P3H2, TP63, NBN, PTEN, BRCA1, and TPX2; the oncogenes EIF4E, CDCA8, MDM2, and PVT1; and the proto-oncogenes SRC, MYC, MCM5, CXCL8, and BCL2. Their deregulation could explain the progression of CC ( Figure 3).

### miRNA binding sites associated with cervical cancer

In 2011, Reshmi *et al.* used BLAT to determine the exact location of four miRNA binding sites associated with CC using bioinformatics programmes and computational tools ^54^. To the best of our knowledge, this study is the first to use BLAT to identify miRNA binding sites in proximity to HPV integration sites involved in CC progression. In this study, 2028 binding sites from 272 CC-associated miRNAs were identified.

Identification of the target mRNAs of these miRNAs is considered a key step in their structural and functional analysis to establish possible interactions and consequently, cellular processes that may be altered in CC progression ^55– 57^. miRNAs located in the two strands of cellular DNA (5’ and 3’ strands) demonstrate their ability to interact in both orientations with the two strands of DNA and form triple helix structures to enhance RNA stability ^58, 59^.

Each CC-associated miRNA showed a different number of binding sites in the human genome ( Table 3, Supplementary File 2), and in the human genomic variants ^17, 21, 60, 61^; miRNAs were distributed throughout the genomes in both intronic or exonic regions ^13^. In this study, CC-associated miRNAs were distributed in the karyosome, with chromosomes 1, 19, 5, 2, 3, 14, 7 and X having the largest number of miRNA binding sites ( Table 4). In order to confirm the distribution of miRNA binding sites, the analysis for each chromosomal following all chromosomes was done. The statistic W Shapiro-Wilk test, show a p-value 0.02; and the mean comparison analysis by ANOVA with a p-value 0.0046 allowed us to confirm the non-random distribution of miRNA binding sites along the genome. These results are consistent with those reported by Calin *et al.* ^12^. Because some chromosomes have a greater number of miRNA binding sites, it provides evidence of a non-random distribution of miRNAs within the chromosomes.

Our results showed a low number of exonic miRNAs. These exonic miRNAs are considered rare miRNAs ^62^, which are important candidates for gaining a better comprehension of interaction networks between miRNAs and their CC-associated targets.

The miRNA binding sites are within a short distance of each other in the chromosome, indicating that they tend to cluster ^63– 66^. Altuvia *et al.* reported miRNAs in groups of two or three ^64^. This coincides with our results on CC-associated miRNA binding sites, as we found that miRNAs are capable of forming groups of more than 6 miRNAs on both strands of human DNA ( Figure 4). We identified an important group of 16 miRNAs that can form these clusters and are located on chromosome 14 region 14q32.31. They include hsa-miR-134, miR-299, miR-323a, miR-329, miR-376a, miR-376c, miR-379, miR-411, miR-485, miR-487a, miR-487b, miR-494, miR-495, miR-539, miR-654 and miR-5095 ( Supplementary File 2). Understanding their individual and collective roles is important when studying the development of this neoplasia.

miR-5095 had the highest number of binding sites distributed throughout the human genome ( Table 3), which is in accordance with previously reported data ^66– 68^ where approximately 900 binding sites were identified; they are probably related to the expression of many target mRNAs and biological processes. Based on its extensive genomic distribution and low specificity in CC, miR-5095 is a good candidate to be used as an indicator of genetic variability within the human population.

### miRNAs located in HPV integration sites

To identify the role of miRNAs, HPV integration sites located in cell cycle-controlling genes were analysed. Thirty-seven miRNAs were identified in HPV integration sites close to cell cycle-controlling genes ( Table 5). Nambaru *et al.* and Schmitz *et al.* identified numerous miRNAs in the proximity of HPV integration sites and reported that approximately 65% of these were involved in cervical carcinogenesis ^8, 9^. Inactivation of tumour suppressor genes by viral integration increases genomic instability and leads to cervical malignant neoplasm progression ^69^.

The multiple miRNA binding sites on a target may decrease the levels of mRNA translation and improve the specificity of gene regulation. For example, one miRNA can have multiple target genes and each individual mRNA can be regulated by numerous miRNAs ^13, 70, 71^. Ninety-seven interactions were identified between miRNAs and cell cycle regulatory genes ( Table 4– Table 5, Figure 4– Figure 6); miR-5095, -548c-5p and -548d-5p showed the highest number of interactions with these kinds of genes.

Ivashchenko *et al.* identified miR-5095 binding sites in the BRCA1 gene ^67^. In this study, miR-5095 was also found to have binding sites in the BAK1, BARD1, CITED2, MDM5, SRC, PARD3B, PPP2CA, RHEB, SOX2 and XPO1 genes ( Table 5 and Figure 6). Our findings provide a basis for searching for other interactions, gene targets, and CC-associated miRNAs.

During miRNA biogenesis, some pre-miRNA produces two mature miRNAs, such as miRNA-5p and miRNA-3p ^72^. Mature miRNA deregulation can have an important role in tumour development, suggesting the need to analyse each mature sequence (miRNA-5p and -3p). In this study, binding sites were analysed for both mature miRNA sequences (-5p and -3p) in several interactions ( Figure 6). A mature miRNA sequence, such as miR-548c, demonstrated binding sites in different cellular genes. Thus, this miRNA could serve as candidate biomarker for CC prognosis and diagnosis.

Han *et al.* characterized the two mature chains of miR-21 and their oncogenic roles in cervical cancer ^73^. The regulation of the mature 5p and 3p chains from several miRNAs has been investigated in other cancers, including colorectal, gastric, breast, lung, kidney, and bladder ^36, 72, 74– 77^, suggesting the need to focus further studies on the two mature chains from the 272 miRNAs reported in this study.

Figure 6 shows the complexity of the interactions between miRNAs and tumour suppressor genes, proto-oncogenes and oncogenes. The study of interaction networks between cell cycle genes and miRNAs involved in cancer is one of the most recent challenges in systems biology and is important for elucidating the control mechanisms for cancer biological process ^78– 81^.

### miRNAs in HPV integration sites and Latin American human genome variants

The differences in miRNA expression profiles between normal and cancerous tissues have led to the identification of clinical biomarkers for the early detection of many diseases, including various cancers and their precursor stages ^79, 82, 83^. Research on miRNAs associated with cancer has not taken into account the genetic variability in human populations, which influences the structure, expression and function of miRNAs in populations from different ethnic backgrounds. Studies on genetic variability are relevant to designing strategies for the diagnosis and prognosis of various diseases.

miR-11-3p, miR-31-3p, miR-107, miR-133a-3p, miR-133a-5p, miR-133b, miR-215-5p, miR-491-3p, miR-548d-5p and miR-944 were conserved in the four human genome variants. In the remaining 27 miRNAs, substitutions, deletions or insertions were observed in the nucleotide sequences, indicating that this variability can be decisive when determining susceptibility to the development of CC ( Table 7 and Supplementary File 3).

There are numerous studies that analyse miRSNPs in different malignancies ^84– 86^, but there is no available data on the correlation of SNPs in CC-associated miRNAs located in HPV integration sites in Latin American human genomic variants.

According to our results, the genomes from Latin America showed a lower miRSNP frequency compared to the control genome (BEB), although the Colombian (CLM) genome frequency was more similar to the BEB genome. Latin American populations have experienced migrations from European, Asian and African individuals ^87^. Thus, our results could be a result of the specific interracial mixing of Colombian populations but also due to migration patterns during human settlement in Latin America.

miRSNPs can affect the structure and function of miRNAs by impacting interactions between miRNAs and their mRNA targets or interfering with the expression levels of individual miRNAs ^20– 22, 88, 89^. miRSNPs could cause the loss or gain of binding sites for the co-evolution of miRNAs and their target mRNA and even influence cell processes related to tumour progression, disease phenotypes or susceptibility to developing a specific disease.

More studies are needed to clarify the role, targets and transcriptional regulatory mechanisms of cellular events in which miRNA are involved, including differentiation, apoptosis, metabolism and carcinogenesis. The expression and deregulation of miRNAs in cancer as well as their role as biological markers in diagnosis and treatment of CC should be explored. Further identification of cellular genes and signalling pathways involved in CC progression could lead to the development of new therapeutic strategies based on miRNAs ^90, 91^. Additional biomarkers associated with apoptosis, necrosis and possible interactions with CRISPR complex sequences from healthy-tumour cervical can be explored in order to develop therapeutic strategies in the future.

## Data availability

The data referenced by this article are under copyright with the following copyright statement: Copyright: ï¿½ 2018 Guerrero Flórez M et al.

Data associated with the article are available under the terms of the Creative Commons Zero "No rights reserved" data waiver (CC0 1.0 Public domain dedication). 

Dataset 1. The mature miRNA reference sequences were obtained in FASTA format from the miRBase database. DOI, 10.5256/f1000research.10138.d164732 ^28^

Dataset 2. Matrix of data containing all the necessary components for the validation of data on CC-associated miRNAs in HPV integration sites in Latin American human genomic variants. DOI, 10.5256/f1000research.10138.d217286 ^36^

## Supplementary material

Supplementary File 1 Articles that mention HPV integration sites, detailing the most frequent types of HPV associated with CC.

Click here to access the data

Supplementary File 2. Diagram indicating the regions on all chromosomes with miRNA binding sites that are associated with cervical cancer.

Click here to access the data

Supplementary File 3. miRNAs identified in HPV integration sites, displaying the nucleotide variations in the selected Latin American human genome variants and in the control variant.

Click here to access the data
