# Supplementary material for: Mapping of microRNAs related to cervical cancer in Latin American human genomic variants
Source: F1000Res. 2018 Dec 5;6:946. Originally published 2017 Jun 20. [Version 2] doi: 10.12688/f1000research.10138.2 (PMC10521080; doi:10.12688/f1000research.10138.2)
Supplement: Peer review history

# Reviewer report: Reviewer response for version 2

**DOI:** 10.5256/f1000research.17592.r47634

**Published:** 2019-04-24

**Authors:** Subhash Mohan Agarwal

**Affiliations:** 1 Bioinformatics Division, ICMR-National Institute of Cancer Prevention and Research, Noida, Uttar Pradesh , India

Is the work clearly and accurately presented and does it cite the current literature?

Yes

If applicable, is the statistical analysis and its interpretation appropriate?

Yes

Are all the source data underlying the results available to ensure full reproducibility?

Yes

Is the study design appropriate and is the work technically sound?

Yes

Are the conclusions drawn adequately supported by the results?

Yes

Are sufficient details of methods and analysis provided to allow replication by others?

No

Reviewer Expertise:

NA

I confirm that I have read this submission and believe that I have an appropriate level of expertise to confirm that it is of an acceptable scientific standard.

# Reviewer report: Reviewer response for version 2

**DOI:** 10.5256/f1000research.17592.r41551

**Published:** 2019-01-03

**Authors:** Juan Manuel Anzola

**Affiliations:** 1 Bioinformatics & Computational Biology, Corporación CorpoGen, Bogotá, Colombia

Most of the comments in my previous review have been addressed. I still find the methodology could have been modified to make more sensitive and more similar to the rules of base pairing of microRNAs. Despite this the conclusions are within reach of the methodology. To me the paper is ready for indexing.

Is the work clearly and accurately presented and does it cite the current literature?

Yes

If applicable, is the statistical analysis and its interpretation appropriate?

Yes

Are all the source data underlying the results available to ensure full reproducibility?

Yes

Is the study design appropriate and is the work technically sound?

Yes

Are the conclusions drawn adequately supported by the results?

Yes

Are sufficient details of methods and analysis provided to allow replication by others?

No

Reviewer Expertise:

NA

I confirm that I have read this submission and believe that I have an appropriate level of expertise to confirm that it is of an acceptable scientific standard.

# Reviewer report: Reviewer response for version 1

**DOI:** 10.5256/f1000research.10920.r24293

**Published:** 2017-09-28

**Authors:** Subhash Mohan Agarwal

**Affiliations:** 1 Bioinformatics Division, ICMR-National Institute of Cancer Prevention and Research, Noida, Uttar Pradesh , India

In the present study the authors have mapped the miRNA involved in cervical cancer on to Latin American genome using in silico predictions. As cervical cancer has the highest mortality rates in low and middle income countries we do need to advance our understanding on mechanism of its progression. It is an interesting study however, there are few shortcomings in the current MS which need to be addressed.

1. It is not clear how human genes near to viral insertion sites have been identified. It was observer that near integration sites mostly only one or two genes are present. The method and parameters used for finding the genes should be detailed so that the results are reproducible. For example have the genes been identified within a particular distance of the insertion sites.
2. Why the authors have mapped the integration sites for 8 types of HPVs collectively and not HPV-16 and 18 alone which are the high risk HPV. Is there any basis for it?
3. The authors have stated that a total of 2028 miRNA binding sites of which 432 were detected in miRBase. In my opinion the analysis should have been restricted to only these sites as they are experimentally identified sites for miRNA binding.
4. As I understand the authors have mapped 42 miRNAs on Latin American genome. It is not clear how 42 miRNAs were selected for this subsequent step.

Minor comments:

1. In the supplementary data the headings of the tables should be in English.
2. Are there 578 or 568 integration sites. It appears from Dataset 2 that there are 568 integration sites. Sheet named "VPH integration sites"
3. Page 4 (last 2 lines) instead of 12 it should be 13. As per the data in figure 3 there are 13 genes in the intermediate category.
4. Methods in Abstract: miRNA sequences associated with CC ……were obtained from miRBase. Shouldn’t it be literature?

Is the work clearly and accurately presented and does it cite the current literature?

Yes

If applicable, is the statistical analysis and its interpretation appropriate?

Yes

Are all the source data underlying the results available to ensure full reproducibility?

Yes

Is the study design appropriate and is the work technically sound?

Yes

Are the conclusions drawn adequately supported by the results?

Yes

Are sufficient details of methods and analysis provided to allow replication by others?

No

Reviewer Expertise:

NA

I confirm that I have read this submission and believe that I have an appropriate level of expertise to confirm that it is of an acceptable scientific standard, however I have significant reservations, as outlined above.

# Response

**Published:** 2018-08-24

**Authors:** Milena Guerrero

**Affiliations:** University of Nariño, Colombia

Dear Reviewer SMA.

 We resubmitted the second version of the paper after addressing the various concerns raised.

 We would like to thank for their time and for their constructive comments to help assist us in improving the manuscript.

 We made the necessary changes in order to address all the specified concerns. The direct responses to the reviewer’s comments are listed below

**Reviewer. SMA**

**Q8**. It is not clear how human genes near to viral insertion sites have been identified. It was observer that near integration sites mostly only one or two genes are present. The method and parameters used for finding the genes should be detailed so that the results are reproducible. For example have the genes been identified within a particular distance of the insertion sites.

**R8**: Thanks for your valuable suggestion. In a previous work (published in Spanish) it was described Guerrero & Guerrero, 2016. We analyze 42 scientific reports including chromosomal bands, HPV genotype, molecular technique for experimental results, and expression profile of miRNA according to lesions in CC.

 Here, we use the description and annotation of genes described in Data Bases, identified positions, regions, chromosomes, sequences and other characteristics to limit the regions of IS. All was manually curated for each chromosome along genome.

**Q9**: Why the authors have mapped the integration sites for 8 types of HPVs collectively and not HPV-16 and 18 alone which are the high risk HPV. Is there any basis for it?

**R9:** Thanks for your valuable comment. In fact, our reason were the results of two of our previous studies about genotyping of HPV in our region, which showed remarkable frequency, in addition to HPV16 and HPV 18, to other as HPV 45, 31, 33, 58, 67, 68. That was the main reason to include these genotypes in our analysis of mapping.

 But also by scientific purposes, because, in the literature is highly frequent to find and to study HPV 16 and 18, but not other genotypes. This information is relevant for us, to understand more depth the natural history of HPV and its mechanisms.

 Sánchez et al., 2013. Published in Spanish

 Nicola N, 2014. Bachelor thesis published in Spanish

**Q10**: The authors have stated that a total of 2028 miRNA binding sites of which 432 were detected in miRBase. In my opinion the analysis should have been restricted to only these sites as they are experimentally identified sites for miRNA binding.

**R10**: Thanks for your valuable comment. To our knowledge, the 2028 binding sites to miRNA and its key role in cervical cancer were identified by the first time in this study, using BLAT mapping. Is an important finding to highlight, compared to 432 binding sites previously reported, and a valuable contribution of bioinformatic tools for this kind research.

**Q11**: As I understand the authors have mapped 42 miRNAs on Latin American genome. It is not clear how 42 miRNAs were selected for this subsequent step.

**R11**: Thanks for your valuable comment. Briefly the pipeline was:

 -BLAT mapping of miRNAs on reference genome

 -Identification of integration sites (IS) of HPV

 -From IS HPV, looking for positions of genes of cell cycle, near to these IS.

 -Functional analysis of IS HPV according to annotations described by Uniprot.

 -With positions of near genes (regulators of cell cycle) and the positions of binding sites of miRNAs, manual mapping for each chromosome was done.

 -Finally, miRNAs in proximity to cell cycle genes control, were identified.

**Minor comments:**

**Q12**. In the supplementary data the headings of the tables should be in English.

**R12**: Thanks for your detailed comment. It was corrected.

**Q13**. Are there 578 or 568 integration sites. It appears from Dataset 2 that there are 568 integration sites. Sheet named "VPH integration sites"

**R13**: Correct. There are 568 HPV integration sites. It was corrected.

**Q14**. Page 4 (last 2 lines) instead of 12 it should be 13. As per the data in figure 3 there are 13 genes in the intermediate category.

**R14**: Correct. Are 13 genes. It is corrected.

**Q15**. Methods in Abstract: miRNA sequences associated with CC ……were obtained from miRBase. Shouldn’t it be literature?

**R15:** Correct. The abstract is re written to include and express data in a clear way.

# Reviewer report: Reviewer response for version 1

**DOI:** 10.5256/f1000research.10920.r23646

**Published:** 2017-07-11

**Authors:** Juan Manuel Anzola

**Affiliations:** 1 Bioinformatics & Computational Biology, Corporación CorpoGen, Bogotá, Colombia

In this work, Guerrero et al. use mature microRNA in order to detect possible targets of these microRNAs in the human genome, and its population variants, including from Latin American, in order to determine possible associations with cervical cancer. 

 I found the paper sound and its results, analysis and conclusions within the reach of the methodology, however I find the methods lacking, in particular when it comes to the parameters used in the BLAT search. BLAT uses a default seed of 11 to do nucleotide searches (they call it tileSize). So it would be good if the authors state clearly what were the BLAT parameters used, in particular "tileSize" and "stepSize". If a 11-word was used for this analysis the authors are running the risk of not being sensistive enough in their searches. High Specificity, Low Sensitivity. It would be interesting to determine how many of the genes reported as being targets for microRNAs are not detected in your search. 

 microRNA have a particular set of rules when it comes to binding to their respective targets, with seeds between 6, 8 or 9 nucleotides. Nothing is stated in the paper to give an idea of how the rules for target detection were used in this paper. See Mullany et al paper.

 It is assumed throughout the paper that all the hits are true positives. There is no measure as to how good is BLAT to detect true vs false positives.

 The paper:

 In your introduction you mention that microRNAs are involved in cancer. The paragraph suggest this is the only role of microRNAs, however they are involved in processes such as development and morphogenesis, so please rephrase this paragraph because cancer is not the only role of microRNAs.

 Figure 7D is better represented as percentage, as in the body of the paper.

 Your phrase: "Because some chromosomes have a greater number of miRNA binding sites, it provides evidence of a non-random distribution of miRNAs within the chromosomes." could be the result of chromosome length. Please provide statistical support for your statement.

 Page 14: Not all Pre microRNAs produce mature ones from both strands, in fact in the great majority of cases is only one strand that produces the mature one.

 The paper will be ready for indexing once these observations are addressed.

Is the work clearly and accurately presented and does it cite the current literature?

Yes

If applicable, is the statistical analysis and its interpretation appropriate?

Yes

Are all the source data underlying the results available to ensure full reproducibility?

Yes

Is the study design appropriate and is the work technically sound?

Yes

Are the conclusions drawn adequately supported by the results?

Yes

Are sufficient details of methods and analysis provided to allow replication by others?

No

Reviewer Expertise:

NA

I confirm that I have read this submission and believe that I have an appropriate level of expertise to confirm that it is of an acceptable scientific standard, however I have significant reservations, as outlined above.

## References

[1] : MicroRNA Seed Region Length Impact on Target Messenger RNA Expression and Survival in Colorectal Cancer. *PLoS One* .2016;11(4) : 10.1371/journal.pone.0154177 e0154177 10.1371/journal.pone.0154177 27123865PMC4849741

# Response

**Published:** 2018-08-24

**Authors:** Milena Guerrero

**Affiliations:** University of Nariño, Colombia

We resubmitted the second version of paper after addressing the various concerns raised.

 We would like to thank you for their time and for their constructive comments to help assist us in improving the manuscript.

 We made the necessary changes in order to address all the specified concerns. The direct responses to the reviewer’s comments are listed below:

**Reviewer. JMA**

**Q1.** I found the paper sound and its results, analysis and conclusions within the reach of the methodology, however I find the methods lacking, in particular when it comes to the parameters used in the BLAT search. BLAT uses a default seed of 11 to do nucleotide searches (they call it tileSize). So it would be good if the authors state clearly what were the BLAT parameters used, in particular "tileSize" and "stepSize". If a 11-word was used for this analysis the authors are running the risk of not being sensitive enough in their searches. High Specificity, Low Sensitivity. It would be interesting to determine how many of the genes reported as being targets for microRNAs are not detected in your search.

**R1**: Thanks for your valuable suggestion. The methodology is re writer. In fact, BLAT only works with tile size 11. This mean that the average total length of mature miRNAs around 16 to 22, and consequently the seed sequence surely is represented at least 50% in mapping with this number of nucleotides.

 About suggestion “to determine how many of genes reports as being for miRNAs are not detected in your search”, we did the search, using programming R. And similar results of reported here we obtained. We not include this new focus on this paper, but If is need, we can send one of R mapping obtained for one chromosome.

**Q2:** microRNA have a particular set of rules when it comes to binding to their respective targets, with seeds between 6, 8 or 9 nucleotides. Nothing is stated in the paper to give an idea of how the rules for target detection were used in this paper. See Mullany et al paper.

**R2**: Thanks for the valuable comment. According to Mullany one of the most important “rules” for binding to mRNA and the role for cancer are length of seed sequence of miRNA. This condition is mentioned in second paragraph of introduction. Despite of this, the analysis no mentioned, but the authors analyzed seed sequences of miRNAs in terms of folding miRNAs (loop, folk, stem), length, 5´UTR extreme, the results was not included for this publication, because is part to another analysis.

**Q3**: It is assumed throughout the paper that all the hits are true positives. There is no measure as to how good is BLAT to detect true vs false positives.

**R3**: Thanks for your valuable annotation. Considering this probability, after that, we use R and bioconductor tools in order to be sure about the mapping results, we found a match between BLAT and R mapping. This data are under analysis ongoing.

**The paper:**

**Q4.** In your introduction you mention that microRNAs are involved in cancer. The paragraph suggest this is the only role of microRNAs, however they are involved in processes such as development and morphogenesis, so please rephrase this paragraph because cancer is not the only role of microRNAs.

**R4**. Correct. The text is re write.

**Q5. **Figure 7D is better represented as percentage, as in the body of the paper.

**R5**. Thanks for the valuable suggestion. The figure was modified, highlighting percentages instead numeric values. File of figures.

**Q6.** Your phrase: "Because some chromosomes have a greater number of miRNA binding sites, it provides evidence of a non-random distribution of miRNAs within the chromosomes." could be the result of chromosome length. Please provide statistical support for your statement.

**R6**: Thanks for the valuable suggestion. The authors included the statistical analysis and confirmed the results, for more clarity the paragraph is re-writing as follow:

 “In order to confirm the distribution of miRNA binding sites, the analysis for each chromosomal following all chromosomes was done. The statistic W Shapiro-Wilk test, show a p-value 0.02; and the mean comparison analysis by ANOVA with a p-value 0.0046 allowed us to confirm the non-random distribution of miRNA binding sites along the genome”.

**Q7.** Page 14: Not all Pre microRNAs produce mature ones from both strands, in fact in the great majority of cases is only one strand that produces the mature one.

**R7**: Thanks for the suggestion. It was adjusted.
